# The prognostic index PRIMA-PI combined with Ki67 as a better predictor of progression of disease within 24 months in follicular lymphoma

**DOI:** 10.3389/fonc.2023.1090610

**Published:** 2023-06-23

**Authors:** Jiaci Hu, Fenghua Gao, Jin Zhao, Wenzhu Song, Yanli Wang, Yuping Zheng, Lieyang Wang, Weie Han, Li Ma, Jingrong Wang, Min Bai, Tao Guan, Yanfeng Xi, Huilai Zhang, Lixia Qiu, Liping Su

**Affiliations:** ^1^ School of Public Health, Shanxi Medical University, Shanxi, Taiyuan, China; ^2^ Department of Hematology, Shanxi Province Cancer Hospital, Shanxi Hospital Affiliated to Cancer Hospital, Chinese Academy of Medical Sciences, Cancer Hospital Affiliated to Shanxi Medical University, Shanxi, Taiyuan, China; ^3^ Department of Lymphoma, Tianjin Medical University Cancer Institute and Hospital, National Clinical Research Center of Cancer, Tianjin, China; ^4^ Department of Pathology, Shanxi Province Cancer Hospital, Shanxi Hospital Affiliated to Cancer Hospital, Chinese Academy of Medical Sciences, Cancer Hospital Affiliated to Shanxi Medical University, Shanxi, Taiyuan, China

**Keywords:** follicular lymphoma, POD24, prognostic index, risk stratification, nomogram

## Abstract

**Background:**

Progression of disease within 24 months (POD24) is a risk factor for poor survival in follicular lymphoma (FL), and there is currently no optimal prognostic model to accurately predict patients with early disease progression. How to combine traditional prognostic models with new indicators to establish a new prediction system, to predict the early progression of FL patients more accurately is a future research direction.

**Methods:**

This study retrospectively analyzed patients with newly diagnosed FL patients in Shanxi Provincial Cancer Hospital from January 2015 to December 2020. Data from patients undergoing immunohistochemical detection (IHC) were analyzed using χ^2^ test and multivariate Logistic regression. Also, we built a nomogram model based on the results of LASSO regression analysis of POD24, which was validated in both the training set and validation set, and additional external validation was performed using a dataset (n = 74) from another center, Tianjin Cancer Hospital.

**Results:**

The multivariate Logistic regression results suggest that high-risk PRIMA-PI group, Ki-67 high expression represent risk factors for POD24 (*P*<0.05). Next, PRIMA-PI and Ki67 were combined to build a new model, namely, PRIMA-PIC to reclassify high and low-risk groups. The result showed that the new clinical prediction model constructed by PRIMA-PI with ki67 has a high sensitivity to the prediction of POD24. Compared to PRIMA-PI, PRIMA-PIC also has better discrimination in predicting patient’s progression-free survival (PFS) and overall survival (OS). In addition, we built nomogram models based on the results of LASSO regression (histological grading, NK cell percentage, PRIMA-PIC risk group) in the training set, which were validated using internal validation set and external validation set, we found that C-index and calibration curve showed good performance.

**Conclusion:**

As such, the new predictive model-based nomogram established by PRIMA-PI and Ki67 could well predict the risk of POD24 in FL patients, which boasts clinical practical value.

## 1 Introduction

Follicular lymphoma (FL) represents one of the common subtypes of indolent non-Hodgkin Lymphoma (NHL) that generally has a good prognosis but is difficult to cure and prone to recurrence (1). Due to different clinical courses, patients may experience multiple relapses and may transition to more aggressive histological types (the current conversion rate for follicular lymphoma is around 2%), or early disease progression, particularly within 24 months of first-line therapy (2–4). Although progression of disease within 24 months (POD24) can currently be used as an early clinical endpoint for short survival of FL patients, there are no simple and efficient predictive markers in clinical diagnosis. As such, how to effectively evaluate POD24 is of great significance (5, 6).

To achieve risk stratification of FL patients, previous studies have proposed a variety of prognostic models, including the classic FL International Prognosis Index FLIPI (7), FLIPI-2 (8), clinical indices-based FLEX models (9) and PRIMA-PI models (10), 23-genes models (11) and m7-FLIPI models (12) developed from genomics models. But given the technical complexity and high cost, there is currently no simple and efficient prognostic model to predict the occurrence of POD24 in patients. Notably, by comparing the ability of FLIPI, FLIPI-2, and PRIMA-PI to identify high-risk patients (POD24) in 475 patients, Stefan et al (13) found that both FLIPI and FLIPI2 significantly overestimated the number of high-risk patients (<50% specificity for 5-year PFS and 5-year OS). In contrast, PRIMA-PI had the highest specificity for identifying high-risk patients (80% for 5-year PFS and 77% for 5-year OS). However, in terms of sensitivity for identifying high-risk patients PRIMA-PI was the least sensitive of the three risk indices (43% for 5-year PFS and 59% for 5-year OS) and FLIPI was the highest (66% for 5-year PFS and 75% for 5-year OS). This demonstrates that the sensitivity of PRIMA-PI in identifying patients with POD24 is not ideal for FL patients initially treated with immunochemotherapy. Notably, PRIMA-PI defines 3 prognostic groups with different clinical courses, and the variability of outcomes within prognostic groups remains high (14). However, it is a powerful and clinically useful prognostic model that can serve as the basis for more complex and comprehensive biomolecular prognostic models. Therefore, current research is focused on integrating clinical and biomarkers to improve predictive power. Based on the simplified PRIMA-PI model, this study combines clinical indicators that are easy to use in conventional practice to explore the relevant risk factors of POD24 in newly diagnosed FL patients and construct a new clinical predictive model, which could provide a theoretical basis for clinicians to carry out more accurate diagnosis stratification and personalized treatment.

## 2 Methods

### 2.1 Patient selection

The data in this study were obtained from a retrospective study of newly diagnosed FL patients (n=135) in Shanxi Cancer Hospital from January 2015 to December 2020. Inclusion criteria: 1) newly diagnosed FL patients diagnosed and graded as per the latest diagnostic criteria of the World Health Organization (WHO) in 2016; 2) patients receiving first-line chemotherapy regimens; 3) patients treated in our hospital for the first time. Exclusion criteria: 1) patients without receiving first-line chemotherapy regimens; 2) patients who await observation or progression of the disease before receiving treatment; 3) Patients with other malignancies or transformations. External validation set was performed in an independent set (n=74) from Tianjin Cancer Hospital, who were treated with R-CHOP (Rituximab, Cyclophosphamide, Doxorubicin, Vincristine, and Prednisone).

The clinical data and immunohistochemical data of the patients in this study were collected through the electronic medical record system of our hospital. Follow-up was conducted by telephone or outpatient, which ended in July 2021. The definition of outcome measures in this study is consistent with other relevant studies (2). POD24 was defined as disease progression or relapse within 24 months after first-line treatment, PFS was calculated from the initial treatment date to the date of death occur via any cause, disease progression or relapse, and OS referred to the time between the date of initial treatment and the patient’s death or last follow-up.

### 2.2 Statistical methods

Previous retrospective cohort studies suggested that (15, 16) the following factors are associated with the prognosis of FL. The first one concerns clinical parameters, including sex, histological grade, extra nodal involvement sites, lactate dehydrogenase (LDH), β2-microglobulin (β2-MG), bone marrow involvement, NK cell percentage, lymphocyte to monocyte ratio (LMR). The second one relates to immunohistochemical indicators, including BCL-2 expression, Ki67 tumor cell expression, MUM-1 expression, and TP53 expression (17–20). The third one emphasizes PRIMA-PI model, which comprises three risk prediction categories: high (β2-MG >3 mg/L), low (β2-MG≤ 3 mg/L, no bone marrow involvement), and median (β2-MG≤ 3 mg/L, bone marrow involvement) (10).

χ^2^ test and multivariate Logistic regression model were used to analyze the risk factors of POD24 in patients undergoing immunohistochemical test, and a predictive model was accordingly constructed. For comparison with the PRIMA-PI model, the AUC, Sensitivity, and Specificity were calculated to explore the accuracy of the model, and the Kaplan-Meier method was used to estimate PFS and OS, which was then compared among the groups with the help of a 2-sided log-rank test. Besides, the least absolute shrinkage and selection operator (LASSO) is a widely used system for regression (21), which was used for the screening of independent variables that affect outcomes and to prevent overfitting. The “glmnet” package was used to generate this regression. A nomogram model was built based on the new predictive model and validated by C-index and calibration curve.

All statistical tests were two-sided, and values of P < 0.05 were considered statistically significant. Statistical analyses were performed using SPSS version 26.0 and R version 4.1.2.

## 3 Results

### 3.1 Patient characteristics

Of the 135 patients with the first-time FL treated with R-CHOP, 20 cases (15%) developed POD24, of which 8 died during the 24-month follow-up, and 92% of patients received maintenance therapy after treatment. In addition, high-grade conversion occurred in 2.9% of patients during the course of treatment. Among the 70 patients treated with R-CHOP, who underwent a full immunohistochemical test (IHC) at the initial diagnosis and were followed up for 24 months, 12 patients (17.1%) developed POD24. The clinical characteristics and molecular index are shown in **Table 1**.

**Table 1:** Clinical and molecular index characteristics [n (%)] and assignments of 70 IHC patients.

Variable	Number	Proportion (%)	Assignment
Gender			
Male	42	60.0	1= Male
Female	28	40.0	2= Female
Number of extra nodal sites			
>=2	52	74.3	1= >=2
<2	18	25.7	2= <2
Histologic grading			
>=3	22	31.4	1= >=3
<3	48	68.6	2= <3
Bone marrow involvement			
Presence	22	31.4	1= Presence
Absence	48	68.6	2= Absence
Ki67 status			
High positive	31	44.3	1= High positive
Low positive	39	55.7	2= Low positive
BCL-2 status			
Positive	61	87.1	1= Positive
Negative	9	12.9	2= Negative
MUM-1 status			
Positive	8	11.4	1= Positive
Negative	62	88.6	2= Negative
TP53 status			
Positive	3	4.3	1= Positive
Negative	67	95.7	2= Negative
LMR level			
Positive	18	25.7	1= Positive
Negative	52	74.3	2= Negative
PRIMA-PI			
High risk	25	35.7	1= High risk
Low/Intermediate risk	45	64.3	2= Low/Intermediate risk
POD24			
Yes	12	17.1	1=Yes
No	58	82.9	2=No

PRIMA-PI, PRIMA-prognostic index; POD24, Progression of disease within 24 months.

### 3.2 Analysis of risk factors for POD24

The χ2 test showed that there were differences in the positive rate of MUM-1, histologic grading, Ki67 expression, and PRIMA-PI risk levels between the two groups of patients with POD24 (P<0.05, **Table 2A**). Patients with POD24 had a high positive rate of MUM-1, high expression of Ki67(the index expression rate is bounded by 30%.), a high risk of PRIMA-PI and a high grading of histologic grading.

Table 2Analysis of risk factors for the occurrence of POD24 in patients.

**Table:** 

A				
Variable	POD24		χ^2^	P
	Yes (12)	No (58)		
**Gender**			0.038	0.846
Male	8(66.7%)	34(58.6%)		
Female	4(33.3%)	24(41.4%)		
**Number of extra nodal sites**			0.007	0.950
>=2	9(75%)	43(74.1%)		
<2	3(25%)	15(25.9%)		
**Histologic grading**			6.488	0.011
>=3	8(66.7%)	14(24.1%)		
<3	4(33.3%)	44(75.9%)		
**Bone marrow involvement**			0.024	0.876
Presence	4(33.3%)	18(31%)		
Absence	8(66.7%)	40(69%)		
**Ki67 status**			7.142	0.008
High positive	10(83.3%)	21(36.2%)		
Low positive	2(16.7%)	37(63.8%)		
**BCL-2 status**			0.188	0.665
Positive	10(83.3%)	51(87.9%)		
Negative	2(16.7%)	7(12.1%)		
**MUM-1 status**			4.502	0.034
Positive	4(33.3%)	4(6.9%)		
Negative	8(66.7%)	54(93.1%)		
**TP53 status**			0.578	0.447
Positive	1(8.3%)	2(3.4%)		
Negative	11(91.7%)	56(96.6%)		
**LMR level**			1.053	0.305
Positive	5(41.7%)	13(22.4%)		
Negative	7(58.3%)	45(77.6%)		
**PRIMA-PI**			7.780	0.005
High risk	9(75%)	16(27.6%)		
Low/Intermediate risk	3(25%)	42(72.4%)

**Table:** 

B					
Variable	β	S. E	Wald	OR (95%CI)	P
Ki67 status	1.940	0.853	5.176	6.958(1.308-37.008)	0.023
PRIMA-PI	1.828	0.763	5.739	6.222(1.394-27.765)	0.017

**(A)** Univariate analysis of POD24 in 70 IHC patients. **(B)** Multivariate logistic regression analysis of POD24 in 70 IHC patients: The variables included in the multivariate logistic regression (stepwise regression back-off method) analysis were Histologic grading, MUM-1 status, Ki67 status, PRIMA-PI. For Histologic grading, P=0.174 (OR=2.854, 95%CI: 0.628-12.961); for MUM-1 status, P=0.337 (OR=2.625, 95%CI: 0.367-18.800) during the screening process.

Significant variables in univariate analysis were included in a multivariate stepwise Logistic regression model (α_in_=0.05 and α_out_=0.1). The results showed that PRIMA-PI risk levels and Ki67 expression represented risk factors for POD24 (P<0.05, **Table 2B**). The risk of POD24 in FL patients with high expression of Ki67 was 6.958 times higher than that of patients with low expression. High-risk PRIMA-PI FL levels were 6.222 times more likely to develop POD24 than low- and medium-risk patients.

### 3.3 Predictive model building and evaluation

The risk factors detected by Logistic regression model and the scoring system established by Ki67 expression were defined as the PRIMA-PIC (termed combined the PRIMA-PI). The model was classified into low-risk group (PRIMA-PI non-high risk, Ki67 low expression); moderate-risk group (PRIMA-PI high risk or Ki67 high expression); high-risk group (high risk of PRIMA-PI, high expression of Ki67).

**Figure 1:**
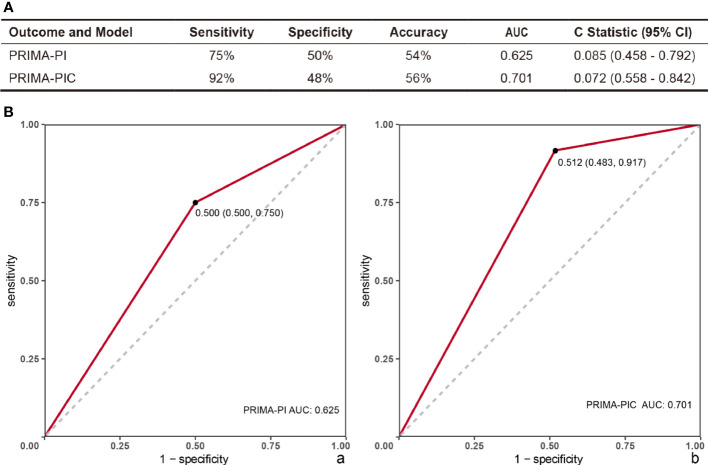
Comparison of the predictive power of PRIMA-PI and PRIMA-PIC. **(A)** Comparison of two models to predict POD24 **(B)** Evaluation model ROC: (a) PRIMA-PI (b) PRIMA-PIC. The comparison results show that PRIMA-PIC has improved.

The Sensitivity and Specificity of the two scoring models in predicting POD24 were evaluated as per the PRIMA-PIC and PRIMA-PI scoring systems in the moderate and high-risk groups (n=41 vs. 38) and the low-risk group (n=29 vs. 32). The results showed that the Sensitivity of PRIMA-PIC in predicting POD24 increased from 75% to 92% of PRIMA-PI, and the Specificity decreased from 50% to 48%, but it was clinically insignificant. Additionally, PRIMA-PIC allowed for the accuracy of predicting POD24 from 54% to 56% (see **Figure 1A**).

The ROC was used to evaluate the PRIMA-PI clinical model and the new model PRIMA-PIC, showing that the AUC of PRIMA-PI was 0.625, with the 95%CI standing at 0.085 (0.458 - 0.792); PRIMA-PIC had an AUC of 0.701, and the 95%CI reached 0.072 (0.558 - 0.842) (**Figure 1B**).

**Figure 2:**
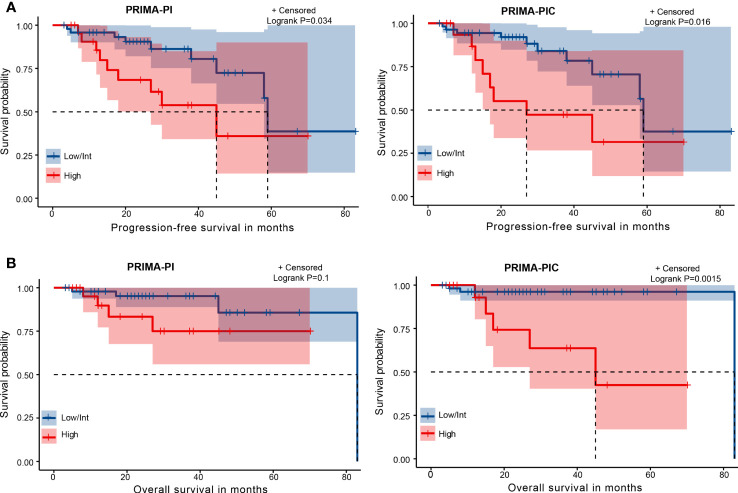
Kaplan-Meier curves for risk groups defined by two scoring systems. **(A)** progression-free survival (PFS) **(B)** overall survival (OS).

We then analyzed Kaplan-Meier curves to estimate the effect of different risk groups on patients’ PFS time and OS time by dividing these 70 patients into the high-risk group and the moderate and low-risk groups according to the PRIMA-PI and PRIMA-PIC scoring systems (**Figure 2**). All the scores could differentiate subgroups of patients with substantially different prognoses. In estimating PFS, PRIMA-PIC (P=0.016) was comparable to PRIMA-PI (P=0.034), while in estimating OS, PRIMA-PIC (P=0.0015) showed better discrimination than PRIMA-PI (P=0.1). Thus, PRIMA-PIC also proved to be a powerful tool for predicting survival.

### 3.4 Construction and validation of the nomogram

**Figure 3:**
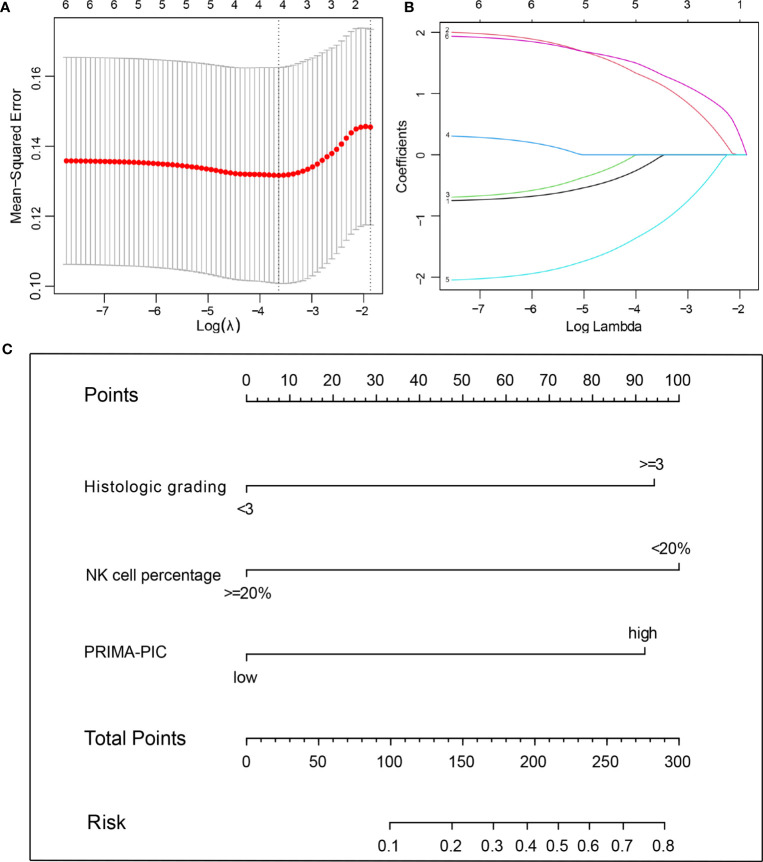
The predictive factors were selected using LASSO regression analysis and nomogram construction. **(A)** Screening for tuning parameter (lambda) in the LASSO regression model. The partial likelihood deviance was calculated as a function of log (lambda), with the least deviance in partial probability corresponding to the optimal number of variables. The dotted vertical lines represented the optimal lambda value based on 1 standard error and the minimum criteria. **(B)** The profiles of LASSO coefficient of the variables of FL patients. The abscissa is the logarithm of lambdas, and the ordinate is the variable coefficient. The variable coefficient continues to decrease as lambdas increase. **(C)** For using the nomogram, place the value ​​assigned to individual patient on each variable axis, and draw an upward line for the determination of the number of points received for each variable value. The sum of these numbers is obtained on the total score axis, and a line is drawn down to the risk axis to calculate the patient’s risk of developing POD24.

The 70 patients were used as the training set, and the other 65 patients were used as the internal validation set. There was no significant difference between them (**Table 3**). In the training set, we used the Lasso regression approach to distinguish risk factors due to the limited sample size and a large number of variables. LASSO regression analysis included the six variables, namely gender, histological grading, number of extra involvement sites, LDH level, NK cell percentage and PRIMA-PIC risk level. The cross-validation and filtering practices of the independent variables are given in **Figures 3A, B**, respectively. The results showed that histological grades, NK cell percentage and PRIMA-PIC risk levels represented risk factors for POD24, based on which, a nomogram model was constructed (**Figure 3C**).

**Table 3:** Comparison of general data of training set and validation set [n (%)].

Variable	All patients (n=135)	%	Training set (n=70)	%	validation set (n=65)	%	P
Gender							0.638
Male	76	56	42	60	34	52	
Female	59	44	28	40	31	48	
Number of extra nodal sites							0.281
>=2	88	65	52	74	36	55	
<2	47	35	18	26	29	45	
Histologic grading							0.501
>=3	46	34	22	31	30	46	
<3	89	66	48	69	35	54	
LDH							0.597
Normal	107	79	55	79	52	80	
Elevated	28	21	15	21	13	20	
NK cell percentage							0.362
Normal	58	43	30	43	28	43	
Elevated	77	57	40	57	37	57	
PRIMA-PIC							0.399
High risk	27	20	15	79	12	18	
Low/Intermediate risk	108	80	55	21	53	52	
POD24							0.128
Yes	20	15	12	17	8	12	
No	115	85	58	83	57	88	

LDH, lactate dehydrogenase; PRIMA-PIC, termed combined the PRIMA-prognostic index; POD24, Progression of disease within 24 months.

**Figure 4:**
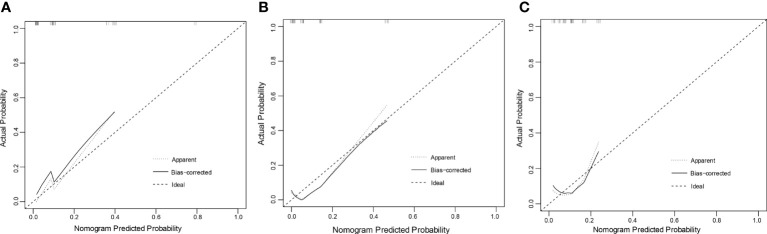
Calibration curve: **(A)** training set **(B)** validation set **(C)** external validation set. The x-axis in the figure represents the probability of POD24 predicted by the nomogram model, and the y-axis represents the actual probability of POD24. The dashed line represents the ideal situation, the oblique angle is 45°, and the solid line represents the correction curve, reflecting the real situation of the nomogram model. The closer the dotted line is to the solid line, the better the model consistency and the better the calibration.

In the training set, the C-index of the nomogram predicting POD24 was 0.869 (0.777-0.959); in the internal validation set, the C-index was 0.856 (0.703-0.998), and in the external validation set, the C-index was 0.716 (0.498-0.934). The calibration curve also showed that the nomogram model established in this study was also well-calibrated (**Figure 4**).

## 4 Discussion

Early detection of high-risk individuals and individualized treatment are necessary to reduce the incidence of POD24 in FL patients (4, 22, 23). Unlike PFS or OS, POD24 is not influenced by other causes (e.g., treatment-related adverse effects) and better reflects the aggressiveness and/or treatment resistance of the disease itself. In this article, the proportion of FL patients who experienced high-grade transformation after treatment was 2.9%, an outcome that is similar to the annual follicular lymphoma transformation rate, and nearly 90% of patients opt for related maintenance therapy after treatment. Studies have identified important clinical markers for the prognosis of POD24, but POD24 reflects the histology of indolent disease, and its prognostic factors may be multifactorial (5, 22). In the past 20 years, experts and scholars worldwide explored various prognostic factors related to FL to accurately predict patients with early disease progression. Currently, Various prognostic models of FL have been proposed, among which the earliest proposed FLIPI and FLIPI-2 models are the most classic prognostic models of FL and the most widely used evaluation systems (24). FLIPI is simple, easy to popularize, and has strong operability, but it cannot reflect the essential characteristics of tumor cells, nor can it be used as a basis for the treatment and choice of treatment in newly diagnosed patients (7). In predicting the prognosis of FL, FLIPI-2 can distinguish groups with significantly different risk of death compared to FLIPI (8), but it also comes with the same drawbacks as FLIPI. The FLEX model based on 9 clinical indicators proposed by Mir et al. (9) can more accurately identify FL patients with poor prognosis, but its ability to identify patients with POD24 is dissatisfactory, with low Sensitivity. In the same year, the gene expression profiling method represented by 23-genes (11) proposed by Huet et al. became a predictive model for FL patients. The lack of clinical validation of prospective studies and the difficulty of reproducing the results make the current use of gene expression profiling to determine the prognosis of FL is limited. The m7-FLIPI (12) prognostic index proposed by Pastore et al, which is also based on gene predictors, includes the mutation status of 7 genes, poor performance status and high-risk FLIPI, enriched by the identification of low-risk patients and high-risk individuals. The study showed that m7-FLIPI had the highest predictive Accuracy for POD24 (2, 12), but it is not suitable for widespread application due to the cost of gene sequencing. Despite their promising prognostics, they are still in the research stage and are limited by the lack of biological or molecular data to enhance clinical predictors. Potential advantages are offset by the high cost and complex techniques of incorporating genetic data into models, so it is important to explore the establishment of cost-effective prognostic indices.

PRIMA-PI (PRIMA-prognostic index) for patients receiving first-line immunochemotherapy regimens, developed by Bachy et al. in 2018, is a simplified clinical index for immunochemotherapy patients based only on β2-microglobulin and bone marrow involvement. We found that the simplified PRIMA-PI was comparable to the classical FL prognostic models FLIPI and FLIPI-2 in prognostic stratification and maintained high specificity in predicting POD24 (10, 13, 25). Therefore, based on the PRIMA-PI clinical prognostic model, this study comprehensively includes the clinical and pathological factors related to the prognosis of FL patients as much as possible to screen out the relevant simple indicators with predictive value.

A total of 4 immunohistochemical detection indicators and 1 surrogate marker were included in this study. Ki67 is a useful proliferation marker in various tumors including lymphoma, Ki67 was determined by immunohistochemical results as nuclear staining, and was classified into 2 levels of high expression (>30%) and low expression (≤30%) based on the percentage of positive cells (18). Usually, Ki67 in IHC is used for routine pathological diagnosis, but Ki-67 has been used as a predictor of early clinical progression and prognosis in NHL as early as the Southwest Tumor Collaborative Group in the United States (26),and Kawaguchi et al. (27) studies have shown that the high expression of Ki67 (>30%) is often associated with shorter overall survival and is a useful marker for patients with high-grade FL in choosing treatment or predicting prognosis. However, there is no relevant study on whether it is related to POD24. This study explored the correlation between Ki67 and POD24 expression and found that the higher Ki67 expression was a risk factor for POD24 (P<0.05). Additionally, it was also found that the positive expression of MUM-1 was also related to POD24, MUM-1 is a nuclear transcription factor expressed mainly in activated post-germinal B cells, immunoblasts, plasma cells and plasma cells, and plays an important role in promoting the differentiation of post-germinal B cells to plasma cells, and its positive expression could be used as an important significance for identifying newly diagnosed FL patients (19). Unfortunately, the parameters included in this study i.e.BCL-2, TP53, and LMR were not shown to be related to POD24. More multi-centre cooperative large-sample clinical trials are needed for further research.

Nomogram model (28) is widely used in cancer prognosis for its ability to quantify predictive models as numerical estimates of event probabilities, with each variable listed separately and each sub-variable as specific points. Afterwards, the cumulative scores (Total points) of all variables are matched with the outcome scale to obtain the predicted probability (29). This is tailored to individual patient characteristics. Its friendly usage and extensive web availability make them popular among oncologists and patients.

Interestingly, it has recently been shown that life stress, an integral part of modern life, can induce tumorigenesis and promote cancer development by modulating genetic and epigenetic changes (30). Stress has the potential power to produce almost all cancer traits in healthy individuals (31), and stress management strategies can restore gene expression through genetic and epigenetic regulation and gene expression as well as therapeutic effects (32). Therefore, whether stressors have an impact on the development of POD24 in follicular lymphoma patients is also an important part of our study to be investigated.

Among the clinical parameters included in the study, PRIMA-PI was consistently shown to be a risk factor for a poor prognosis for POD24 (33). FLIPI-1, FLIPI-2 and PRIMA-PI were not significantly different in predicting POD24, and relatively PRIMA-PI had the best specificity, but its sensitivity was relatively weak (5). The results of our study showed that, PRIMA-PIC, a new model that incorporates Ki67 into PRIMA-PI, found that the Sensitivity and Accuracy of predicting POD24 using the new model were better compared with PRIMA-PI. Yet, the Specificity was similar. There is currently no valid prognostic model for POD24. Compared with molecular models such as m7-FLIPI and 23-genes, PRIMA-PIC is more efficient and convenient. As such, the model constructed in this study has practical significance.

In conclusion, high-risk PRIMA-PI level, high expression of Ki67, and positive MUM-1 represent prognostic risk factors for POD24, which could help clinicians to better diagnose and treat patients. In addition, we can see from the study that PRIMA-PIC is also an effective tool for predicting survival. It can better identify high-risk patients in predicting PFS time and OS time in patients with follicular lymphoma. However, this study also has certain limitations: firstly, since it’s a retrospective study, it may be subjected to retrospective bias and follow-up bias. Secondly, in recent years BR and R-CVP treatments have been widely used to treat FL patients, but because the data set of this study is a retrospective study from previous years, the number of patients using both treatment regimens did not meet statistical criteria and were excluded in the inclusion exclusion criteria. However, some patients with R-CVP were used in the study of the PRIMA-PI model, so further analysis of the dataset for this group of patients is needed. Also, whether this model can be used in patients under BR treatment needs to be further explored and analyzed. On the other hand, the sample size is limited, the prognostic factors we chose to include were based on some of the most influential factors in domestic and foreign studies. It should be noted that there are other factors that influence outcomes that were not included in our study. These may prove to have some value in predicting the risk of POD24 in FL patients, and the results of the Specificity of the two models are different from those of other studies. Therefore, a large-sample, multi-centre randomized controlled study was needed to study the correlation between the relevant immunohistochemical indicators and outcomes shown in the study.

## Data availability statement

The original contributions presented in the study are included in the article/supplementary material. Further inquiries can be directed to the corresponding authors.

## Ethics statement

The study was reviewed and approved by the Research Ethics Committee of Shanxi Hospital, Cancer Hospital Chinese Academy of Medical Sciences (grant NO. 2022233).

## Author contributions

Contribution: LS, LQ, JH contributed to conception and design. JH, FG, JZ, YW, YZ, LW, WH, LM, JW, MB, TG, YX, HZ, LQ, LS took part in collection and assembly of clinical data. JH, FG, JZ, WS were responsible for data analysis and interpretation. JH, WS, LQ, LS involved in manuscript writing. LS, LQ, HZ participated in study supervision. All authors listed have made a substantial, direct, and intellectual contribution to the work.
